# Neonatal Proteinuria in Calves—A Quantitative Approach

**DOI:** 10.3390/ani11123602

**Published:** 2021-12-20

**Authors:** Wiesław Skrzypczak, Alicja Dratwa-Chałupnik, Małgorzata Ożgo, Karolina Boniecka

**Affiliations:** Department of Physiology, Cytobiology and Proteomics, West Pomeranian University of Technology Szczecin, Klemensa Janickiego 29, 71-270 Szczecin, Poland; alicja.dratwa-chalupnik@zut.edu.pl (A.D.-C.); malgorzata.ozgo@zut.edu.pl (M.O.); karolina.boniecka@zut.edu.pl (K.B.)

**Keywords:** neonatal period, calves, proteinuria, albumin, low and high molecular weight proteins

## Abstract

**Simple Summary:**

A newborn’s survival depends on the quick adaptation of the organism to new environmental conditions. Newborn calves show high somatic maturity compared to, for example, human newborns, but their body functions with a lower efficiency than that of adult cattle. Adaptation processes concern all organs, including the kidneys, which are not morphologically mature after birth. The ongoing morphological changes imply functional alterations in the kidneys. There is an increase in blood flow through the kidneys and the glomerular filtration rate, as well as an increase in the efficiency of resorption processes of primary urine components into the blood, including proteins. Protein is present in trace amounts in the urine of healthy adults of various species. It can occur in larger amounts in the urine of sick animals, as well as in certain physiological states, e.g., in newborns. The presence of protein in the urine of newborns in the quantity exceeding 300 mg/24 h/m^2^ is called neonatal proteinuria. The causes of proteinuria in healthy newborns have not been clearly elucidated. Many studies have focused on proteinuria in newborns and sick children and sick animals, especially dogs and cats. The present study was the first to quantify the hypothesis of the occurrence of proteinuria in healthy calves in the first week of life and to assess its intensity and dynamics, based on the analysis of changes in total protein excretion in the urine and its fractions differing in molecular weight (albumin, low molecular weight proteins (LMW) and high molecular weight proteins (HMW)). It should be noted that the analysis of excreted protein fractions is a sensitive diagnostic indicator of the type of kidney disease, e.g., increased HMW protein excretion may indicate damage to the renal glomeruli, and loss of LMW proteins may indicate renal tubular disease.

**Abstract:**

Urine testing is a convenient, non-invasive method of obtaining information about body functions. Depending on the intended purpose, urine testing may be qualitative and/or quantitative. Urine analysis can also include proteins. There are no data in the literature on the occurrence of proteinuria in healthy neonatal calves. The present study was the first that aimed to quantify the hypothesis of proteinuria occurrence in these animals in the first week of life, to assess its intensity and dynamics and to understand the underlying causes of proteinuria in healthy calves. The research was carried out on 15 healthy calves in the first seven days of life. Calves were catheterized to determine minute diuresis. Total protein concentration was determined in blood plasma and urine. Urine proteins were separated by electrophoresis (SDSPAGE) and their concentration and percentage were determined by densitometry using an image archiving and analysis software. The separated proteins were divided into three groups according to molecular weight for albumin, LMW and HMW proteins. The results were standardized per 1 m^2^ of body surface area and statistically analyzed. Neonatal proteinuria was demonstrated in healthy calves, mainly resulting from the high concentration of LMW proteins in the urine. Their percentages decreased significantly from 84.46% on the first day of calves’ life to 64.02% on day 7. At the same time, a statistically significant increase was observed in the proportion of albumin and high molecular weight proteins in urine total protein. Albumin percentage increased from 9.54% (on day 1) to almost 20% (on day 7), while the proportion of HMW proteins increased from 6.68% to 18.13%, respectively. The concentration of total protein in the urine of newborn calves amounted to 14.64 g/L and decreased statistically significantly during the first 72 h of postnatal life, stabilizing at the level of 3–4 g/L. The mean value of total protein excretion in the first week of life was 4.81 mg/min/m^2^ (i.e., 6.93 g/24 h/m^2^). The analysis of protein concentration in the urine and its excretion, as well as changes in urinary excretion of the tested protein fractions, indicated that neonatal proteinuria in healthy neonatal calves was tubular (i.e., main reason is the reduced absorption of proteins in nephrons). In addition, research showed that there was a rapid improvement in resorptive mechanisms in tubular cells. It should be assumed that the filtration barrier in the kidneys of these animals after birth is morphologically prepared to retain high molecular weight proteins. It seems that the increased permeability of the filtration barrier in the glomeruli does not necessarily indicate the immaturity of the kidneys, but may indicate the kidneys’ adaptation to excess protein removal from the body during feeding with high-protein food (colostrum), with an open intestinal barrier enabling protein absorption from the gastrointestinal tract to the blood.

## 1. Introduction

Urine testing is a convenient, non-invasive method of obtaining information about body functions. Depending on the intended purpose, urine testing may be qualitative and/or quantitative. Urine analysis can also include proteins. It is known that protein in the urine of healthy individuals of different species is present in trace amounts. It can occur in larger quantities in the urine of sick animals, as well as in certain physiological states, e.g., in newborns [1,2,3,4,5].

Proteinuria, even in healthy individuals, is an indicator of renal dysfunction, and the type of the excreted protein fraction (albumin, low molecular weight proteins (LMW) or high molecular weight proteins (HMW)) may be a marker of the type of kidney disease, e.g., albumin and higher molecular mass protein excretion may indicate glomerular damage, while loss of LMW proteins may indicate tubular diseases [6].

The main cause of proteinuria observed in newborns of various species is morphological immaturity of the kidneys and dynamic adaptive processes occurring especially in the first days postpartum, associated with both changes in renal hemodynamics and nephron function [4,7,8,9,10,11,12,13,14,15,16].

Most of the proteins excreted in the urine are plasma proteins that have been excreted into the urine due to increased permeability of the filtration barrier or decreased tubular resorption. Albumin molecule size is assumed to be a limiting value for the filtration of plasma proteins. As the particle size decreases, the ease of their filtration in the glomeruli is also higher. Elevated protein filtration is also caused by increased blood protein concentration, increased blood flow through the kidneys, increased filtration pressure in the glomeruli and electrostatic charge of proteins [1,17].

The resorption of filtered proteins occurs mainly in the convoluted part and in the initial straight part of the proximal tubules. The efficiency of protein absorption and hydrolysis mechanisms in tubular cells determines (apart from filtration) the intensity of proteinuria. Therefore, the determination of individual protein fractions in the urine of newborns may be a sensitive diagnostic tool in assessing the maturity and functioning of the filtration barrier and resorption mechanisms in the proximal tubules [1,18].

It should be noted, however, that the causes of neonatal proteinuria, mechanism, frequency and dynamics have not been clearly explained. Many studies have focused on proteinuria in newborn and sick children and animals, especially dogs and cats [4,11,13,19,20,21]. There are no data in the literature on the occurrence of proteinuria in neonatal calves. 

The aim of the research was to verify the hypothesis about the occurrence of proteinuria in healthy calves in the first week of life and assess its intensity and dynamics based on the following tests: (a) total protein concentration in the urine, (b) percentage of albumin and LMW and HMW proteins in the urine, (c) excretion of protein and individual fractions in the urine and (d) percentage of albumin, LMW and HMW proteins in 24 h urine. The analysis of these indices in the urine produced over a 24 h period in consecutive days of the first week of life will enable not only quantitative but also qualitative evaluation of proteinuria, i.e., distinguishing between glomerular and tubular proteinuria. It will also help to understand and elucidate the underlying causes of proteinuria in newborn calves.

## 2. Materials and Methods

### 2.1. Animals 

The research was carried out on 15 healthy Polish-Friesian var. Black-and-White full-term female calves in the first seven days of life. Directly after birth calves were fed with their dam’s colostrum, and then animals were transported to the animal facility of the Department of Animal Physiology and placed in individual stalls, under environmental conditions ensuring welfare maintenance. During the research period animals, were subject to constant observation and showed no clinical symptoms of diseases. During the first two days of life, calves were fed their mothers colostrum in total amount up to 4 L (4–5 feeds per 24 h). In the following days of the first week of their life, animals were fed mother’s milk 3 times per day, in a total amount of 5 L in the 3rd day of life and in an amount of 6–7 L per day up to the end of the experiment. As stated above, the animals were fed individually with their mother’s colostrum and milk. According to that, the composition of the colostrum and milk have not been analyzed. The first feeding of the day and weighing of the calves took place one hour before the start of the study. The analyses started in the morning at 9:00. At first, the calves had a Foley balloon catheter inserted into the bladder. Urine was collected at two 30 min time intervals into sterile polyethylene bags in order to determine minute diuresis. Blood for tests was collected from the external jugular vein via the catheter inserted at the first time of the blood sampling 15 min after the beginning of the urine collection intervals and protected against clotting with heparin (Heparin–Biochemie, Langkampfen, Austria). 

### 2.2. Analyses

Blood and urine, immediately after collection, were centrifuged at 3000 and 2000 rpm, respectively, for 15 min at 4 °C. The concentration of total protein in the blood plasma and urine was determined by the modified Lowry method (Bio-Rad DC Protein Assay) using the bovine serum albumin standard. Urine samples (1 mL) were lyophilized for concentration. The obtained urine lyophilisate was dissolved in 100–150 μL of deionized water before performing protein electrophoretic separation. Urine samples were mixed with Laemmli buffer containing sodium dodecyl sulfate (SDS–Fluka Chemie GmbH, Buchs, Switzerland) and reducing agent 2-mercaptoethanol (βME–Merck KGaA, Darmstadt, Germany), and subsequently denatured for 5 min at 95 °C.

Electrophoretic separation of urine proteins was carried out on a 15% SDS-polyacrylamide gel, SDS PAGE. Their concentration was determined by densitometry using the Bio Print 215 image documentation and analysis system and the Vilber Lourmat Bio-1D software. Molecular weight markers (Broad Range SDS-PAGE Molecular Weight Standards, Bio Rad, Hercules, CA, USA) were used to estimate the molecular weights of separated protein fractions with the following composition: Myosin 200.00 kDa, β-galactosidase 116.25 kDa, Phosphorylase b 97.40 kDa, Serum albumin 66.20 kDa, Egg albumin 45.00 kDa, Carbonic anhydrase 31.00 kDa, Trypsin inhibitor 21.50 kDa, Lysozyme 14.40 kDa, Aprotinin 6.50 kDa.

The separated proteins were visualized in a staining solution with the following composition: 0.05 g of Coomassie Brilliant Blue R 250 (Fluka Chemie GmbH, Buchs, Switzerland), 1 mL of methanol, 50 mL of 10% acetic acid and 450 mL of distilled water. The gels were subsequently archived with a GS-800 densitometer (Bio-Rad) and analyzed using the Quantity One software (Bio-Rad, Hercules, CA, US). The investigated protein bands were divided into three groups: albumin (BSA, bovine serum albumin) and proteins with lower (LMW < 69 kDa) and higher molecular weight (HMW > 71 kDa). The concentration of selected fractions of proteins in the urine was calculated on the basis of optical density, and their percentage in the total urine protein was calculated. In addition, the following were calculated: urinary total protein excretion rate (UV_TP_), urine volume low molecular weight proteins (UV_LMW_), urinary albumin excretion rate (UV_alb_), urinary high molecular weight protein excretion rate (UV_HMW_), as well as 24 h urinary protein excretion and the percentage of LMW proteins, albumin and HMW in 24 h urine (U, concentration of the component in urine; V, diuresis rate (mL/min)).

The results were standardized per 1 m^2^ body surface area according to the Meeh formula:

$$P=0.105\cdot \sqrt[{3}]{bw.^{2}}$$

where *bw* is body weight (kg).

### 2.3. Statistical Analysis

Mean values and standard deviations were calculated. The obtained results were subjected to statistical analysis in order to assess the significance of differences using the method of univariate analysis of variance with repeated measurements using Duncan’s multiple range test (Statistica 6.0 software).

## 3. Results

**Figure 1:**
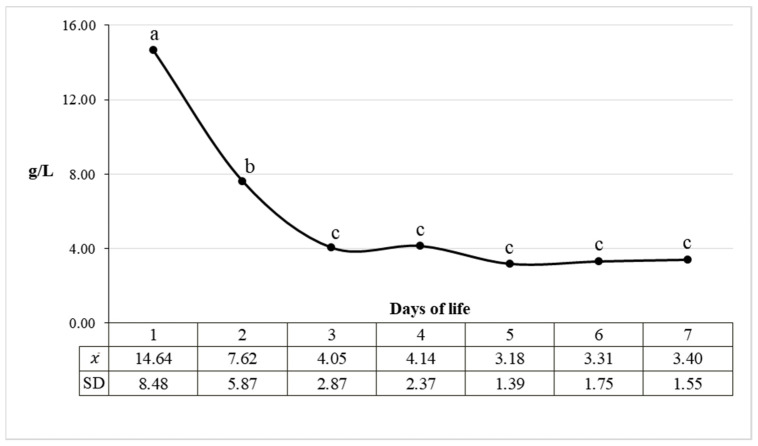
Mean urine total protein concentration in calves during the first 7 days of life. Letters a, b and c mark the significant differences at *p* ≤ 0.05 between the values of urine total protein concentration. Values marked with different letters differ significantly.  χ—mean value, SD—standard deviation.

The concentration of total protein in the urine of newborn calves on the first day of life amounted to 14.64 g/L and decreased statistically significantly during the first 72 h of postnatal life, stabilizing at the level of 3–4 g/L (Figure 1). Individual variability in the value of this parameter was observed, especially in the first three days of life. 

**Figure 2:**
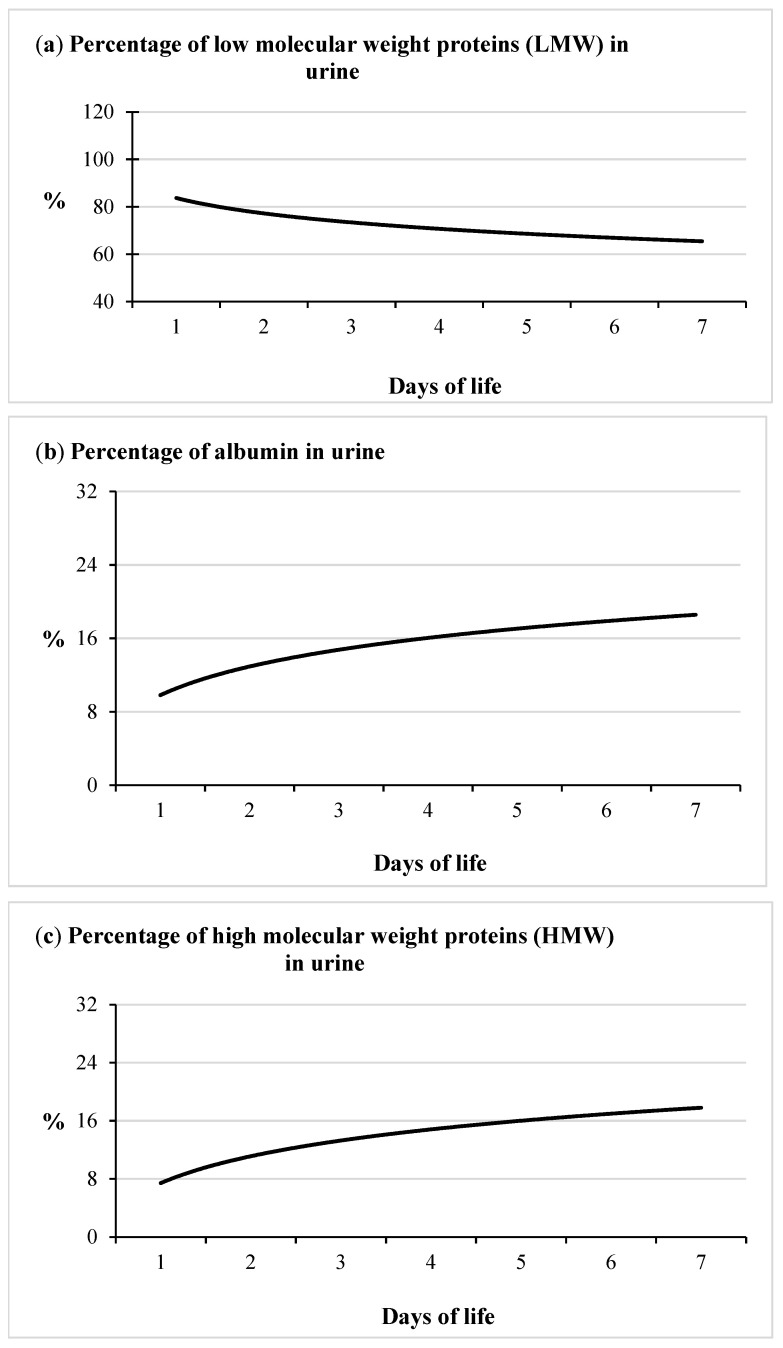
The percentage of urinary protein fractions: LMW (**a**), albumin (**b**) and HMW (**c**) in calves (the continuous curve represents the tendency line for this parameter). The detailed data concerning the information about the percentage of protein fractions distribution are presented in Table 1.

Dynamic changes in the proportion of individual protein fractions in the urine were demonstrated. Low molecular weight (LMW) proteins constituted the highest percentage in urine total protein. Their percentages decreased significantly, from 84.46% on the first day of life to 64.02% on day 7 (Figure 2a, Table 1). At the same time, a statistically significant increase was observed in the proportion of albumin and high molecular weight proteins in urine total protein. Albumin percentage increased from 9.54% (on day 1) to almost 20% (on day 7) (Figure 2b, Table 1), while the proportion of HMW proteins increased from 6.68% to 18.13% in the same period (Figure 2c, Table 1).

**Figure 3:**
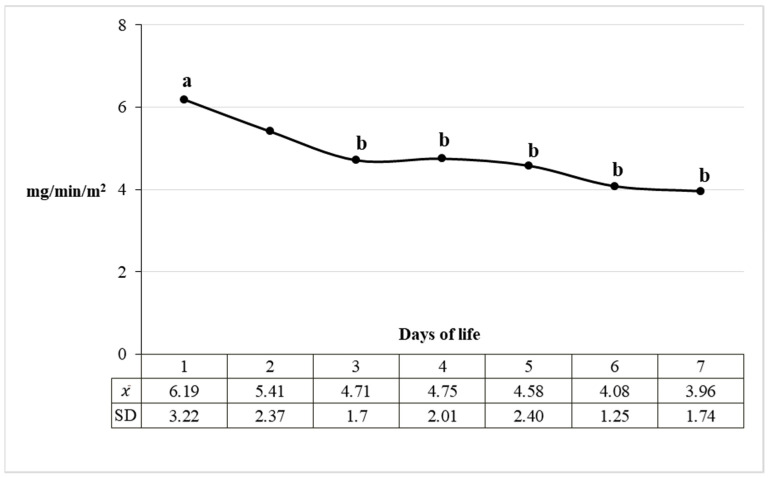
Mean urinary excretion of total protein in the first 7 days of life in calves per body surface area. Letters a and b mark the significant differences at *p* ≤ 0.05 between the values of protein excretion with urine. Values marked with different letter differ significantly. χ—mean value, SD—standard deviation.

**Figure 4:**
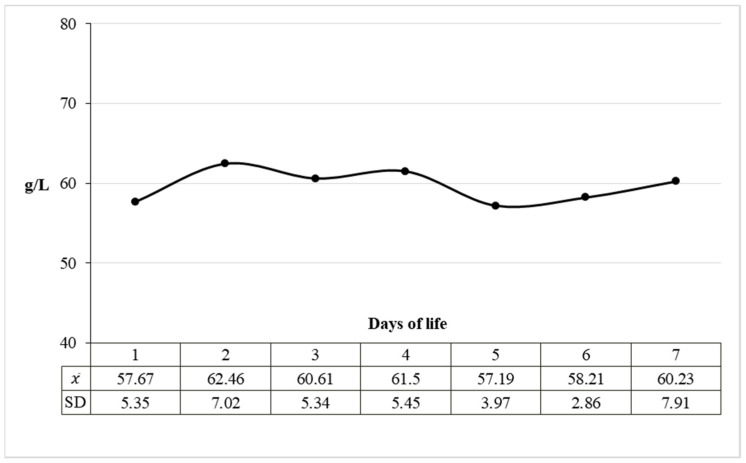
Mean blood plasma total protein concentration in calves during the first 7 days of life. *χ*—mean value, SD—standard deviation.

A statistically significant reduction in the total protein excretion rate in the urine was demonstrated, especially in the first three days of life of calves. Urinary total protein excretion rate was 6.19 mg/min/m^2^ body surface area on the first day of life and 3.96 mg/min/m^2^ body surface area on day 7 (Figure 3). The daily excretion of total protein in the urine calculated on this basis decreased and in the consecutive days of the first week of life amounted to 8.91, 7.79, 6.78, 6.84, 6.59, 5.87 and 5.70 g/24 h/m^2^ BSA. It is noteworthy that despite the dynamic processes of protein excretion in the urine, its concentration in the blood was stable (Figure 4).

**Figure 5:**
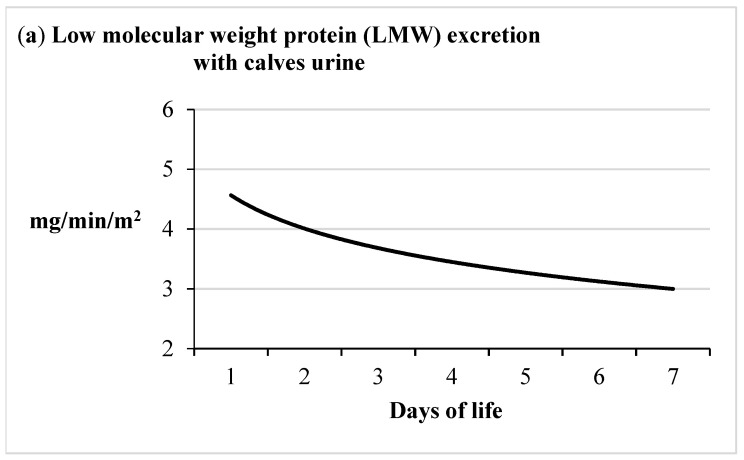

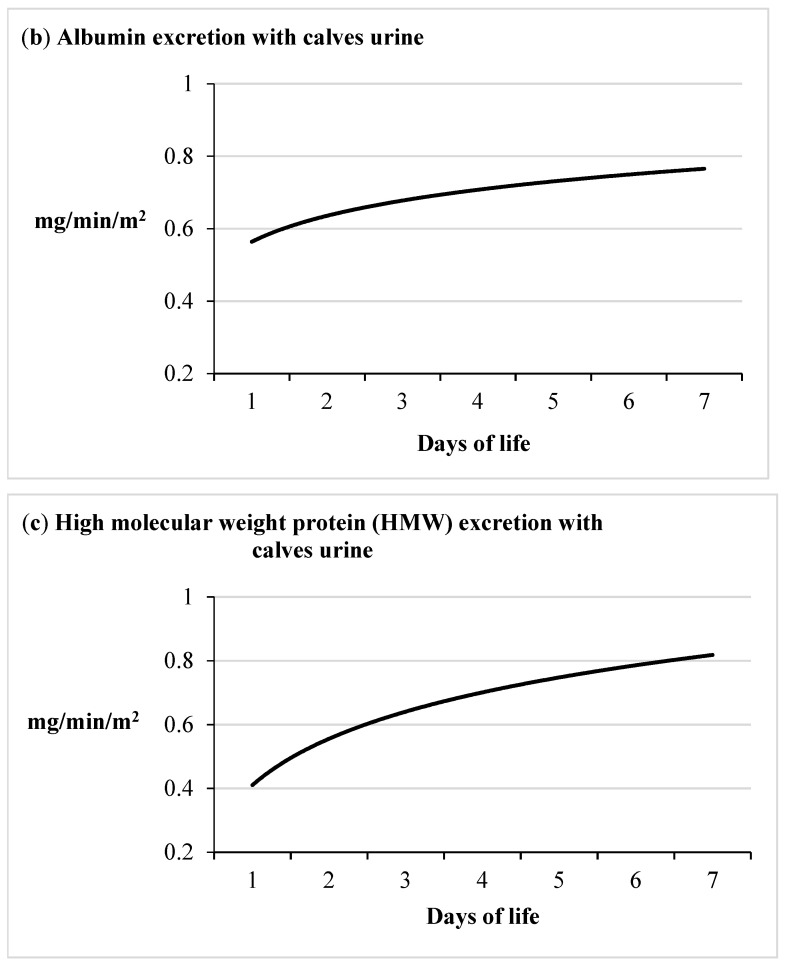
Mean urinary excretion of LMW, albumin and HMW proteins in the first 7 days of life in calves per body surface (the continuous curve represents the tendency line of this parameter). The detailed data concerning the information about the protein excretion with urine are presented in Table 1.

The decrease in total protein excretion in the urine was a result of a significant reduction in the excretion of LMW proteins, with an increase in the excretion of albumin and HMW proteins (Figure 5, Table 1). The excretion of low molecular weight proteins decreased statistically significantly, from 4.30 (on the first day of life) to 2.52 mg/min/m^2^ body surface area (on the seventh day). Albumin excretion tended to increase and amounted to 0.50 mg/min/m^2^ body surface area (on the first day of calves’ life) and 0.69 mg/min/m^2^ body surface area (on the seventh day of life). The excretion of high molecular weight proteins increased more than twofold, from 0.31 (on the first day of life) to 0.69 mg/min/m^2^ body surface area (on the seventh day).

**Figure 6:**
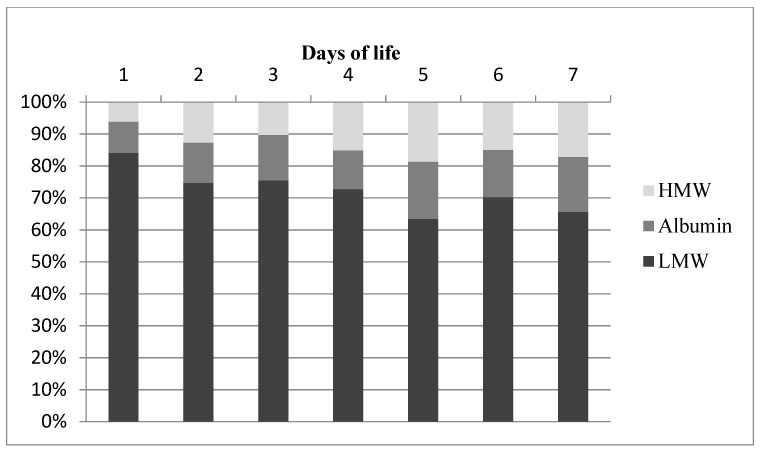
Percentage of low molecular weight protein (LMW), albumin and high molecular weight proteins (HMW) in daily urine of calves.

The proportions of individual protein fractions in 24 h urine are presented in Table 1 and Figure 6. LMW proteins had the highest percentage and their content ranged from 63.48% to 84.10%. It was the highest on the first day of life, exceeding 84%. The proportion of albumin in 24 h urine increased with age and amounted to 9.78% on the first day and 17.65% on the seventh day. The total percentage of LMW proteins and albumin (which are relatively easily filtered into primary urine) in 24 h urine of calves was the highest and amounted to 93.88% on the first day after birth and 81.32% on the fifth day of life. The percentage of HMW proteins in 24 h urine did not exceed 19%. It was the lowest on the first day of life (6.12%) and highest on the fifth day (18.68%). 

## 4. Discussion

Proteinuria occurs in healthy newborns of many animal species and humans; however, high species and individual variability in the frequency, duration and 24 h quantity of excreted proteins is observed, especially in the first days after birth [3,8,11,14,21,22,23,24].

The amount of protein excreted in the urine is primarily the result of glomerular filtration of proteins and their resorption in the proximal tubules. Morphological changes in nephrons occurring in the postnatal period imply functional alterations in the newborns’ kidneys. Test results for the kidney function of many neonates, including calves, indicate that renal blood flow and glomerular filtration are low at this stage of life. With age, an increase in these parameters is observed, as well as the resorptive and secretory efficiency of the renal tubules [3,4,7,8,12,16,23,25,26,27,28].

Moreover, birth age and body weight are important for the occurrence of neonatal proteinuria [8,20,23,28,29,30,31,32,33,34]. It has been shown that the amount of protein excreted in the urine may be inversely correlated with gestational age [4,13].

In the present experiment, neonatal proteinuria was observed in healthy full-term calves, along with a trend towards a decrease in total protein content in the urine during the first week of life. Dynamic changes in the percentage of individual protein fractions in the urine occurring at this time should be emphasized. LMW proteins constituted over 80% of all proteins in the urine of newborn calves, and this proportion decreased by 20% within one week. A convergent trend of changes in the proportion of LMW proteins was demonstrated by Ożgo et al. [14] in the urine of young goats. The authors reported that the percentage of proteins with a molecular weight lower than 69 kDa in the urine of male kids decreased from 87.4% (on day 1) to 80.9% (on day 7) and 74.8 (on day 30). The high percentage of this protein fraction in the urine of various newborn species was confirmed by the results of other authors.

A significant increase was noted in albumin percentage in the urine of calves in the first 72 h after birth (from 9.54% to 15.07%). In view of the stabilized protein concentration in the blood plasma of calves, the cause of the observed changes could be a higher GFR resulting in an increase in the filtered protein load in the glomera and/or less efficient tubular resorption. In studies performed on newborn goats, an increase in the proportion of albumin in the urine was also observed in the first days of life, from 7.26% on the first day to 12.25% on the second day and 12.05% on the third day [22]. The direction of changes in albumin concentration in the urine in the first days of life, i.e., high concentration in the first day and decreasing in the following days, was observed in human newborns compared to that observed in calves [1]. According to Utsch and Klaus [35], a low albumin fraction in the urine could indicate a non-renal cause of proteinuria.

The tendency of increasing proportion of HMW proteins in the urine of calves demonstrated in the experiment was confirmed by the results of the studies conducted by [22], who observed an increase in the proportion of this protein fraction in male kids’ urine in the first week of life, from 5.36% to 11.42%. The percentage of HMW proteins in young male goats’ urine increased in the following weeks of life, but it did not exceed 20% on day 30.

There are no unambiguous data in the literature concerning the quantity of proteins excreted in the urine of newborn animals, including calves, and the trends of changes in the excretion of total protein and individual protein fractions. The study carried out on male kids showed a reduction in the total protein excretion rate in the urine from 3.82 mg/min/m^2^ (on day 1) to 1.65 mg/min/m^2^ (on day 7) [22]. These results confirmed the trend of changes in total protein excretion observed in calves, while the amount of protein excreted in the urine of young male goats was about two times lower than in the urine of calves. A reduction in urinary protein excretion in the first days after birth was also found in newborns of other species, while the values of urinary protein excretion varied [3,6,10,11,19]. The above data confirm the existence of interspecific differences in urinary protein excretion. 

The species differences were also indicated by trends in the excretion of individual protein fractions. The decrease in LMW protein excretion observed in the first week of calves’ life was confirmed by other authors. Ożgo et al. [14] showed a significant (*p* < 0.01) reduction in the excretion of LMW proteins in the urine of newborn kids from 3.41 g/min/m^2^ BSA (on day 1) to 1.33 g/min/m^2^ BSA (on day 7). The authors believe that the reduction in LMW protein excretion in the urine of young male goats observed in the first week of life, despite the changes in the concentration of these proteins in the blood plasma and increasing glomerular filtration, indicated an increasing efficiency of the tubular mechanisms of protein resorption in the kidneys of newborns. Joseph and Gattineni [4] reported that the function of the proximal tubules in a healthy neonate’s kidney was often not sufficiently mature to prevent leakage of smaller proteins; therefore, newborns’ urine showed an increased concentration of proteins compared to adult urine.

In the first week of calves’ life, an upward trend in the urinary albumin excretion rate was observed, while in kids, a highly significant (*p* ≤ 0.01) increase in the urinary albumin excretion rate was recorded only in the first 24–48 h of life (from 0.26 to 0.43 mg/min/m^2^ BSA). From the third to the seventh day of life, a decrease in albumin excretion to the value of 0.12 mg/min/m^2^ BSA was observed [22]. According to the authors, the presented trends of changes indicated a quick and effective tightening of the filtration barrier.

The increasing excretion tendency of HMW proteins with the urine of calves shown in the present experiments was confirmed by the results of studies on newborn young goats, in which an increase in the excretion of this protein fraction was observed in the first three days of life, from 0.156 to 0.277 g/min/m^2^ [22]. The authors indicated that this could be related to the increased permeability (immaturity) of the filtration barrier for these proteins. According to these authors, the reduction in the excretion rate of this protein fraction in the urine from day 3 to 5 and stabilization at the level of approximately 250 mg/min/m^2^ BSA indicated effective tightening of the filtration barrier. It should be noted that these authors observed a re-increase in HMW protein excretion from the third week of life to a value of 573 mg/min/m^2^ BSA 30 days after birth. High postpartum excretion of HMW proteins was observed in human newborns, as well as a decrease in this fraction excretion in the following days, which, according to the authors, was evidence of tightening of the filtration barrier. A decreasing excretion of selected HMW proteins was also observed in young dogs in the first days of life [11].

Other authors also drew attention to the increased permeability of the filtration barrier for proteins and suggested that the maturation of the filtration barrier in the postnatal period preceded absorption system maturation in the nephrons [4,6].

The daily excretion of protein in the urine of calves (from 5.7 to 8.91 g/m^2^ BSA) allowed us to classify neonatal proteinuria in these animals as significant, and the predominance of the LMW protein fraction in the urine proved that it was selective proteinuria. The study carried out on young male goats also showed the presence of selective proteinuria and a reduction in total protein excretion in the urine, but the 24 h amount of protein excreted was lower than in calves (from 2.38 to 5.50 g/24 h/m^2^). The daily excretion of individual protein fractions ranged from 1.92 to 4.92 g/24 h/m^2^ (LMW proteins), from 0.16 to 0.43 g/24 h/m^2^ (albumin) and from 0.22 to 1.07 g/24 h/m^2^ (HMW proteins). Joseph and Gattineni [4] reported that the normal 24 h protein excretion in full-term human neonates was 68–309 m g/24 h/m^2^. Higher values indicate the presence of proteinuria.

## 5. Conclusions

The research has shown the presence of neonatal proteinuria in healthy calves. It was associated with a high concentration of proteins with a molecular weight of ≤70 kDa. Albumin and proteins with lower masses constituted over 85% of all urine proteins. The mean value of total protein excretion rate in the first week of life was 4.81 mg/min/m^2^ (i.e., 6.93 g/24 h/m^2^). The highest protein concentration in the urine was recorded on the first day of life and it significantly decreased in the first three days. The urinary protein excretion rate showed a similar direction of change. The results indicated that reduced tubular absorption was the cause of high urinary protein concentration and increased excretion in the first days of life. Therefore, neonatal proteinuria in healthy newborn calves was tubular in nature. In view of the stable protein concentration demonstrated in the blood plasma, the decrease in urine protein concentration and stabilization at the level of about 4 g/L from day 3–4 of life indicated a rapid improvement of the resorptive mechanisms in the renal tubular cells. This was confirmed by the decreasing tendency of the percentage content and LMW protein excretion, with an increasing, albeit small, proportion of albumin and HMW proteins in the urine. The low excretion rate of HMW proteins in the first days of life (0.31 mg/min/m^2^) and the low percentage of total urine protein (6.68% on day 1) indicated that the filtration barrier in the kidneys of calves was morphologically prepared for the retention of proteins of high molecular weight.

## Figures and Tables

**Table 1:** Mean urinary percentages, urinary excretion and percentage daily urinary excretion of protein fractions.

				Days of Life						
				1	2	3	4	5	6	7
Percentage in urine (%)			LMW	84.46 ^a,c^	75.07 ^b,c^	74.29 ^b,c^	72.22 ^b,c^	66.38 ^b,c^	69.65 ^b,c^	64.02 ^b^
			Albumin	9.54 ^a,c^	13.27 ^b,c^	15.07 ^b,c^	15.27 ^b,c^	19.03 ^b^	15.37 ^b,c^	19.51 ^b^
			HMW	6.68 ^a,c^	13.05 ^b,c^	11.88 ^b,c^	14.45 ^b,c^	18.04 ^b^	15.18 ^b,c^	18.13 ^b^
Urinary excretion (mg/min/m^2^)			LMW	4.30 ^a^	4.15 ^a^	3.87 ^a^	3.80 ^a^	3.14 ^a^	3.32 ^a^	2.52 ^b^
			SD	2.49	1.93	1.92	2.02	1.98	1.81	1.64
			Albumin	0.50 ^a,c^	0.70 ^c^	0.73 ^b,c^	0.63 ^a,c^	0.88 ^b^	0.70 ^a,c^	0.69 ^a,c^
			SD	0.11	0.34	0.35	0.27	0.45	0.32	0.33
			HMW	0.31 ^a,c^	0.71 ^b,c^	0.53 ^c^	0.79 ^bc^	0.92 ^b^	0.71 ^b,c^	0.69 ^b,c^
			SD	0.23	0.73	0.43	0.5	0.63	0.46	0.26
Percentage of protein fractions in daily urine excretion [%]			LMW	84.1 ^a^	74.66 ^a^	75.47 ^a^	72.74 ^a^	63.48 ^b^	70.19 ^b^	67.40 ^b^
			Albumin	9.78 ^a,c^	12.61 ^c^	14.23 ^b,c^	12.10 ^a,c^	17.84 ^b^	14.83 ^a,c^	17.65 ^a,c^
			HMW	6.12 ^a,c^	12.73 ^b,c^	10.30 ^c^	15.16 ^b,c^	18.68 ^b^	14.98 ^b,c^	17.65 ^b,c^

LMW—low molecular weight proteins, HMW—high molecular weight proteins, SD—standard deviation. ^a–c^—different letters of the alphabet indicate significant differences at *p* ≤ 0.05 between the values of respective protein fractions in the first week of life on different days.

## Data Availability

The data presented in this study are available on request from the corresponding author.
